# Association between dietary acid load and the odds of ulcerative colitis: a case–control study

**DOI:** 10.1038/s41598-023-41069-6

**Published:** 2023-08-23

**Authors:** Mina Movahedian, Hadi Emamat, Hadith Tangestani, Samaneh Rashvand, Hamid Ghalandari, Mohammad Hossein Somi, Azita Hekmatdoost

**Affiliations:** 1 Student Research Committee, Department of Clinical Nutrition and Dietetics, Faculty of Nutrition and Food Technology, National Nutrition and Food Technology Research Institute, Shahid Beheshti University of Medical Sciences, Tehran, Iran; 2 The Persian Gulf Tropical Medicine Research Center, The Persian Gulf Biomedical Sciences Research Institute, Bushehr University of Medical Sciences, Bushehr, Iran; 3 Department of Nutrition, Faculty of Health and Nutrition, Bushehr University of Medical Sciences, Bushehr, Iran; 4 Department of Clinical Nutrition and Dietetics, Faculty of Nutrition and Food Technology, National Nutrition and Food Technology, Research Institute Shahid Beheshti University of Medical Sciences, P.O. Box: 19395-4741, Tehran, Iran; 5 Department of Clinical Nutrition, Faculty of Nutrition and Food Sciences, Shiraz University of Medical Sciences, Shiraz University of Medical Sciences, Shiraz, Iran; 6 Division of Gastroenterology, and Hepatology, Tabriz University of Medical Sciences, Tabriz, Iran

**Keywords:** Gastroenterology, Health care, Signs and symptoms

## Abstract

Ulcerative colitis (UC) is one of the two types of inflammatory bowel disease (IBDs), which have a pivotal role in weakening the quality of lives of suffering patients. According to some recent studies, significant changes in dietary patterns may have contributed to the increased prevalence of UC. Potential renal acid load (PRAL) is an index used to estimate dietary acid load of the diet. The aim of the current study is to investigate the association between PRAL and odds of UC. The current case–control study included 62 newly diagnosed cases of UC and 124 healthy controls. Dietary habits of participants in the last year were collected with a valid food frequency questionnaire (FFQ). Thereafter, PRAL score was calculated based on a formula containing the dietary intake of protein, phosphorus, potassium, calcium, and magnesium. Participants were categorized according to quartiles of PRAL. Multivariable logistic regression models were used to estimate the odds' ratio (OR) with 95% confidence intervals (CIs) of UC across quartiles of PRAL. The results of the current study indicated that in the crude model, participants in the fourth quartile of PRAL had 2.51 time higher odds of UC compared with those in the first quartile of the PRAL [(OR 2.51; 95% CI 1.03–6.14), (P = 0.043)]. After adjustment for age and biological gender, this positive association remained significant [(OR 2.99; 95% CI 1.16–7.72), (P = 0.023)]. In the final model, after further adjustment for BMI, current smoking, education, Helicobacter pylori infection, and dietary intakes of total energy, omega-3 fatty acids, trans-fatty acids, and total dietary fiber, the odds of UC in the highest quartile of PRAL was significantly higher compared to the lowest quartile [(OR 3.08; 95% CI 1.01–9.39), (P = 0.048)]. So, we observed that higher dietary acid load assessed by PRAL score is associated with greater odds of UC.

## Introduction

Inflammatory bowel diseases (IBDs) are chronic, relapsing inflammatory disorders of the gastrointestinal (GI) tract, characterized by abdominal pain and diarrhea. There are two main phenotypes of IBDs, namely ulcerative colitis (UC) and Crohn's disease (CD). These disorders drastically affect the quality of life of suffering patients^1^. The prevalence of UC is increasing all over the world^2^. Moreover, UC-related morbidity and mortality, health care, and societal costs are significant^3^. Various factors are involved in the pathophysiology of UC, including genetics, environmental factors, immune system disorders, and epithelial barrier defects^4^. It has been suggested that remarkable changes in dietary patterns during the past decades might play a role in the increased prevalence of UC^5,6^.

It has been hypothesized that dietary factors play a pivotal role in the initiation and development of UC^7–9^. Several dietary patterns, such as Western dietary regimen, dietary patterns rich in animal proteins and animal products, those with high content of saturated fats or altered ratios of omega-6 to omega-3, and those low in fruits and vegetables have been postulated to contribute to increased intestinal inflammation^10–13^. Therefore, it can be deduced that changing the dietary pattern in individuals with active IBDs, from an animal-based diet to a predominantly plant-based diet, might result in the inhibition of intestinal inflammation, reduction of disease severity, and maintaining the clinical remission^10^. In contrast, following a Western dietary approach rich in animal meat, dairy products, fat, simple sugars, processed meats, alcohol, and restricted in vegetables and fruits, has been shown to be associated with an increased risk of developing IBDs^14,15^. Evidence also suggests that following the Mediterranean dietary pattern is significantly related to improved clinical condition and reduction of inflammatory markers^14,16,17^. Furthermore, there exists abundant evidence that the individual’s dietary composition can affect the acid–base balance of the body^18,19^. For instance, it has been claimed that consumption of potassium and magnesium (provided mainly by the intake of fruits and vegetables) may be associated with a more alkaline environment in the human body. On the contrary, consuming a western diet is usually associated with increased acidity^20,21^. Based on studies, the kidneys remove the products of metabolism of some anions (chlorine, phosphorus and sulphate), organic acids and cations (sodium, potassium, calcium, magnesium). If the amount of anions exceed the cations, the urinary acid excretion mechanism (hydrogen ion H+) is stimulated^22^. Potential renal acid load (PRAL) is an index used to estimate dietary acid load of the diet^23^. In other word, PRAL is a capacity of acid or base production of any food, which includes the amount of endogenously synthesized organic acids^22^. The concept of PRAL calculation is physiologically based and takes under consideration the diverse rates of intestinal absorption of sulfur-containing minerals and proteins, as well as the sum of sulfate produced from metabolized proteins^24^. Therefore, PRAL is an index calculated from the sum of proteins and phosphorus as an anion, minus some cations such as potassium, calcium and magnesium^25^. A lower PRAL indicates a more alkaline diet which results in an elevated 24-h urinary pH^26^. If the body fails to maintain the acid–base balance, dietary acid load can cause metabolic acidosis^21^.

Several experimental studies have postulated that metabolic acidosis can lead to tissue damage and, consequently, initiate inflammatory responses^27–29^. Human studies have also indicated such an association between acid load and inflammatory status of the body. For instance, Wu et al. demonstrated that a higher dietary acid load (i.e. a higher PRAL) is associated with the increased levels of C-reactive protein (CRP) (an important inflammatory marker) in breast cancer survivors^21^. Different studies have been conducted to investigate the association between the dietary acid load and the progression of various disorders, such as non-alcoholic fatty liver disease (NAFLD)^30,31^, diabetes mellitus^32^, hyperuricemia^26^, albuminuria and impaired kidney function^33^, breast cancer^21^, and osteoporosis^34^. Nonetheless, the association between dietary acid load and the incidence/recurrence of IBDs has not been investigated. Due to this vacancy of information, the present study was designed to investigate the association between PRAL and UC.

## Methods

### Participants

The present case–control study was conducted on newly diagnosed UC patients (< 6 months) in a referral hospital during the year 2013, Tabriz city, Iran. The details of study protocol have been published elsewhere^35^. Briefly, sixty two newly diagnosed patients (< 6 months) with UC were recruited as the case group. Inclusion criteria for the cases included: individuals recently diagnosed with UC, based on patients’ signs and symptoms and their colon tissue pathology report. The exclusion criteria for the case group included history of any other gastrointestinal diseases, carcinoma, and other inflammatory, infectious, and autoimmune disorders. One hundred and twenty-four age- and sex-matched healthy subjects were enrolled in the study as the control group. The control group was selected from individuals without UC visiting orthopedic clinics of the same referral hospital. The individuals suffering from gastrointestinal illnesses/symptoms (gastro-esophageal reflux disease (GERD), diarrhea, irritable bowel syndrome (IBS), and abdominal pains) or conditions which might have caused drastic changes of dietary habits (i.e. metabolic diseases, including diabetes mellitus, cardiovascular diseases (CVDs), gout, and hyperlipidemia) were excluded from the control group. The age range for the two groups was 20–80 years.

Participants completed an informed consent prior to enrollment. Information about demography, medical history, body mass index (BMI), drugs, dietary intake, current smoking, *Helicobacter pylori* infection, education level, and familial history of UC were obtained through completing the general information questionnaire. The study protocol was approved by the ethics committee of the Shahid Beheshti University of Medical Sciences (ethical code: IR.SBMU.RETECH.REC.1401.723. All methods were performed in accordance with the relevant guidelines and regulations.

### Dietary assessment and calculation of potential renal acid load (PRAL)

Dietary habits of participants in the last year were collected by a trained interviewer with a valid and reliable 168 items food frequency questionnaire (FFQ)^36^. Then PRAL score was calculated based on the dietary intake of protein, phosphorus, potassium, calcium, and magnesium using the following formula^25^:

$$ {\text{PRAL }}\left( {{\text{mEq}}/{\text{day}}} \right) \, = \, \left( {{\text{Protein }}\left[ {{\text{g}}/{\text{day}}} \right] \, \times \, 0.{49}} \right) \, + \, \left( {{\text{Phosphorous }}\left[ {{\text{mg}}/{\text{day}}} \right] \, \times \, 0.0{37}} \right) \, {-} \, \left( {{\text{Potassium }}\left[ {{\text{mg}}/{\text{day}}} \right] \, \times \, 0.0{21}} \right) \, {-} \, \left( {{\text{Calcium }}\left[ {{\text{mg}}/{\text{day}}} \right] \, \times \, 0.0{13}} \right) \, {-} \, \left( {{\text{Magnesium }}\left[ {{\text{mg}}/{\text{day}}} \right] \, \times \, 0.0{26}} \right) $$

### Statistical analysis

The data was analyzed using Statistical Package for Social Sciences software (version 20.0; SPSS, Chicago, IL, USA). Kolmogorov–Smirnov test was used to evaluate normality. The qualitative and quantitative variables were compared between groups using the chi-square test and independent t-test or one-way ANOVA test, respectively. Participants were categorized according to quartiles of PRAL (Q1: − 59.49 to − 9.87, Q2: − 9.45 to − 0.28, Q3: − 0.08 to 7.79, Q4: 7.80 to 41.51). We used multivariable logistic regression models to estimate the odds ratio (OR) of UC across quartiles of PRAL. In all models, the first quartile was determined as the reference. In logistic regression analysis, UC and PRAL were considered as the dependent and independent variables, respectively. The ORs and 95% confidence intervals (CIs) were reported. Possible confounding factors, including gender (male/female), age (years), BMI (Kg/m^2^), current smoking (yes/no), education (Primary, Secondary and high school or university education), Helicobacter pylori infection (yes/no), and dietary intakes of total energy (kcal/day), omega-3 fatty acids (g/day), trans-fatty acids (g/day), and total dietary fiber (g/day) were adjusted in the final model. P-values < 0.05 were considered as statistically significant.

### Ethics approval and consent to participate

The study protocol was approved by the ethics committee of the Shahid Beheshti University of medical sciences (ethical code: IR.SBMU.RETECH.REC.1401.723.). Written informed consent was signed by all subjects participated.

## Results

General characteristics and dietary intakes of participants across quartiles of PRAL are presented in Table 1. Sixty-two recently newly diagnosed UC patients and 124 healthy controls were included in the present study. The mean ± standard deviation (SD) of age and BMI of all subjects were 36.63 ± 12.42 years and 25.39 ± 3.82 kg/m^2^, respectively. The frequency of male sex was significantly different between the quartiles of PRAL (P = 0.026). Also, total calories, total proteins, total fat, saturated fatty acids (SAFA) and monounsaturated fatty acids (MUFA) were significantly different across quartiles of PRAL (P < 0.05). Regarding other general characteristics, there was no significant difference between PRAL quartiles.

**Table 1:** General characteristics and dietary intakes of participants according to the quartiles of PRAL.

	Quartiles of PRAL				
	Q1(n = 47)	Q2 (n = 46)	Q3 (n = 47)	Q4 (n = 46)	P-value^a^
Age (years)	37.80 ± 12.8	36.97 ± 13.1	39.02 ± 12.4	32.65 ± 10.5	0.07
Male (%)	29.8	37	48.9	58.7	0.026
Body mass index (kg/m^2^)	25.82 ± 3.8	25.39 ± 3.7	25.81 ± 3.5	24.52 ± 4.1	0.31
Education (%)					0.70
Primary	10.6	8.7	6.4	2.2	
Secondary and high school	55.3	47.8	53.2	52.2	
University	34	43.5	40.4	45.7	
Current Smoking (%)	6.4	15.2	8.5	4.3	0.27
*H. pylori* infection (%)	0	6.5	4.3	6.5	0.36
Family history of UC (%)	2.1	0	2.1	0	0.57
Total calories (Kcal/day)	2584.3 ± 598.7	2573.1 ± 661.6	2623.7 ± 542.8	3001.3 ± 592.8	0.001
Total carbohydrate (g/day)	366.5 ± 82.1	350.5 ± 99.9	353.1 ± 82.2	380.6 ± 87.3	0.33
Total protein (g/day)	81.1 ± 18.5	84.6 ± 27.3	90.2 ± 20.5	114.6 ± 31.2	< 0.001
Total fat (g/day)	95.2 ± 30.2	98.0 ± 27.5	99.6 ± 26.9	117.5 ± 30.6	0.001
SAFAs (g/day)	27.2 ± 9.86	27.8 ± 9.32	29.5 ± 11.7	34.4 ± 10.5	0.004
MUFAs (g/day)	31.8 ± 10.2	33.6 ± 9.77	33.2 ± 9.16	40.1 ± 10.9	0.001
PUFAs (g/day)	23.1 ± 8.62	23.7 ± 7.93	22.9 ± 7.82	26.3 ± 6.90	0.13
Trans-fatty acids (g/day)	0.027 ± 0.011	0.031 ± 0.007	0.010 ± 0.002	0.026 ± 0.007	0.57
Total dietary fiber (g/1000 kcal)	20.91 ± 6.84	21.97 ± 8.33	20.14 ± 7.30	22.24 ± 8.06	0.52

Data are presented as mean (standard deviation) for continuous variables and percent for categorically distributed variables.

^a^Chi-square test for qualitative variables and one-way ANOVA test for quantitative variables.

*PRAL* potential renal acid load, *UC* ulcerative colitis.

Dietary intakes of participants in the case and control groups are presented in Table 2. Dietary intake of total calories, carbohydrate, fat, protein, SAFA, MUFA, polyunsaturated fatty acid (PUFA), phosphorus, potassium, and magnesium were significantly higher in cases (P < 0.05). The range and mean ± SD of cases and controls pertaining to PRAL scores were − 59.49 to 41.51 and − 0.24 ± 14.76 mEq/day, respectively.

**Table 2:** Dietary intakes of cases and controls.

	Case (n = 62)	Controls (n = 124)	*P* value^a^
Total calories (kcal/day)	2902.3 ± 643.3	2590.8 ± 585.2	0.001
Total carbohydrate (g/day)	381.54 ± 86.25	353.25 ± 88.05	0.039
Total fat (g/day)	113.81 ± 34.95	96.92 ± 25.46	0.001
SAFAs (g/day)	32.92 ± 11.83	28.19 ± 9.78	0.004
MUFAs (g/day)	39.17 ± 12.73	32.43 ± 8.27	< 0.001
PUFAs (g/day)	26.02 ± 9.06	23.01 ± 7.08	0.024
Trans-fatty acids (g/day)	0.0135 ± 0.01	0.0119 ± 0.01	0.17
Total dietary fiber (g/1000 kcal)	20.19 ± 6.92	21.87 ± 7.94	0.15
PRAL, mEq/day	2.57 ± 15.24	− 1.66 ± 14.37	0.06
Protein (g/day)	100.79 ± 33.43	88.49 ± 23.82	0.011
Phosphorus (mg/day)	1648.26 ± 461.52	1516.48 ± 399.63	0.046
Potassium (mg/day)	3847.82 ± 959.57	3588.36 ± 993.67	0.09
Calcium (mg/day)	1173.44 ± 372.58	1153.67 ± 353.37	0.72
Magnesium (mg/day)	434.79 ± 103.19	399.27 ± 106.77	0.032

Data are presented as mean ± standard deviation.

^a^Independent sample t-test test.

*PUFA* polyunsaturated fatty acid, *MUFA* monounsaturated fatty acid, *SAFA* saturated fatty acid, *PRAL* Potential renal acid load.

The ORs (95% CI) of UC across quartiles of PRAL are shown in Table 3. In the crude model, participants in the fourth quartile of PRAL had 2.51 time higher odds of UC compared with those in the first quartile of the PRAL [(OR 2.51; 95% CI 1.03–6.14), (P = 0.043)]. After adjustment for age and biological gender, this positive association remained significant [(OR 2.99; 95% CI 1.16–7.72), (P = 0.023)]. In the final model, after further adjustment for BMI, current smoking, education, *Helicobacter pylori* infection, and dietary intakes of total energy, omega-3 fatty acids, trans-fatty acids, and total dietary fiber, the odds of UC in the highest quartile of PRAL was significantly higher compared to the lowest quartile [(OR 3.08; 95% CI 1.01–9.39), (P = 0.048)].

**Table 3:** The OR (95%CI) of UC across quartile categories of PRAL.

	Dietary potential renal acid load						
	Q1(n = 47)	Q2 (n = 46)	P-value	Q3 (n = 47)	P-Value	Q4 (n = 46)	P-value^a^
Cases/controls	11/36	18/28		13/34		20/26	
Range of PRAL mEq/day	− 59.49 to − 9.87	− 9.45 to − 0.28		− 0.08 to 7.79		7.80 to 41.51	
Model 1*	1.00 (Ref)	2.10 (0.85–5.16)	0.10	1.25 (0.49–3.17)	0.63	**2.51 (1.03–6.14)**	**0.043**
Model 2^†^	1.00 (Ref)	2.18 (0.88–5.41)	0.09	1.29 (0.50–3.32)	0.59	**2.99 (1.16–7.72)**	**0.023**
Model 3^‡^	1.00 (Ref)	2.41 (0.85–6.86)	0.09	1.70 (0.57–5.11)	0.33	**3.08 (1.01–9.39)**	**0.048**

Significant values are in bold.

^a^Multivariable logistic regression test.

*Crude model.

^†^Adjusted for age and gender.

^‡^Additionally adjusted for body mass index, current smoking, education, *Helicobacter pylori* infection, total energy intake, omega-3 fatty acids, trans-fatty acids, and total dietary fiber.

*PRAL* potential renal acid load.

## Discussion

In the present case–control study, we observed that there is a positive association between dietary acid load assessed by PRAL score and the development of UC. After adjusting for confounding variables, our results showed that subjects in the highest quartile of PRAL were approximately 3 times more likely to develop UC than those in the lowest quartile.

Diet is one of the main determinants of the acid load in the body, which must excreted by the kidneys^37^. In general, foods such as cheese, meat, eggs and grains create an acid load, whereas fruits and vegetables provide alkali in the body. Based on this, plant-based dietary patterns are considered to be mainly reducing, and on the contrary, animal-based dietary patterns are considered to be mainly increasing the acid load or PRAL in the body^38^.

To the best of our knowledge, there has been no study investigating the association between the dietary acid load and the risk of IBDs. Nonetheless, research indicates a positive correlation between animal-based diets and the likelihood of developing IBDs, whereas plant-based dietary approaches have demonstrated a negative association with the probability of being affected by IBDs^39^. In line with the findings of the current study, a large prospective cohort study involving 125,445 participants and a 14-year follow-up period revealed an 11% increased risk of developing UC, associated with a dietary pattern that includes both white and red meat varieties^40^. In another cohort study with even larger scales, it was observed that higher intake of total meat and red meat increases the risk of UC by 40% and 60%, respectively^41^. Likewise, Limketkai et al. showed that, compared to a Western diet, following a plant-based diet can reduce active symptoms of the UC by 70%^42^.

Some mechanisms have been proposed to justify the association between various dietary patterns and IBDs. Amongst these, higher total antioxidant capacity (TAC) provided by a diet composed mainly of plant-based foodstuff seems likely to play a role in reducing the risk of UC^43^. Moreover, recent studies propose that changes of the intestinal microbiota might be imperative in the pathophysiology of IBDs^44^. On this note, the prebiotic effects of a diet rich in insoluble fibers, leading to increased production of short-chain fatty acids (SCFAs) that can modify gut microbial composition, have also been proposed as an additional mechanism by which this dietary approach may influence the risk of IBDs^45,46^. On the other hand, an animal-based regimen is believed to negatively affect the diversity and composition of gut microbiota, paving the way for a more IBD-inducing environment^47^. Furthermore, the production of toxic metabolites by intestinal bacteria, such as hydrogen sulfide (H_2_S) produced from bacterial metabolization of unabsorbed sulfur-containing amino acids, can compromise the integrity of intestinal integrity, leading to impaired barrier function of colonic epithelial cells. These metabolites have also been postulated to interfere with the proliferation of these cells and, thus, hinder their regeneration. These pathways might eventually incentivize an already susceptible environment towards colonic abnormalities, including UC^48,49^.

The end-products obtained from metabolizing animal-based foodstuff tend to produce a net acidic content, as opposed to more alkaline metabolites produced from plant-based foodstuff^23^. Chronic dietary acidic conditions in the body, accompanied by changes in the body's buffering capacity, eventually lead to a low-grade metabolic acidosis, inflammation, and cell and tissue damage^23,50^. Studies on human subjects also indicate a link between dietary acid load and inflammation. For instance, Wu et al. showed that breast cancer survivors who are in the highest dietary acid load quartile have 30–33% higher plasma levels of C-reactive protein (CRP)^21^. Likewise, another study showed that PRAL had a significant correlation with TNF-α levels^51^. It is worthy of note that prolonged inflammatory state of the intestinal mucosa has been postulated to be one of the main factors in the pathogenesis of UC^52^, presenting another link between an animal-based regimen and the pathophysiology of the disease. In addition, studies have found that dietary acid load can also exert adverse metabolic effects by increasing blood levels of cortisol^19^; on the same note, it has been observed that cortisol levels are higher in more severe cases of IBDs^53^. These observations propose additional justifications for the presumed association between PRAL and UC.

To the best of our knowledge, the association between dietary acid load and the odds of UC has not been previously investigated through a well-designed case–control study. Also, the study was conducted in Iran as a developing country where cultural and socio-economic factors play major roles in creating significant inter-individual variances in dietary intake. However, this study had some limitations. Firstly, the case–control design of the study cannot ensure a causal relationship; further investigations, especially in the format of cohort and interventional studies, are needed to certify the directionality of the observed association. Secondly, although the FFQ questionnaire used in the present study has been validated, it is inherently subject to recall bias and measurement errors. Also, the limitation of the sample size did not allow us to create sex-specific quartiles. Finally, more objective measurements, including biomarkers, such as serum levels of inflammatory markers and electrolytes and the composition of intestinal microbiota are suggested to be taken into consideration in future studies.

## Conclusion

In the current study, it was observed that higher dietary acid load assessed by PRAL score is associated with greater odds of UC. We recommend that future prospective studies with long-term follow-up periods be conducted to further clarify the causal relationship between dietary acid load and the risk of UC.

## Data Availability

The datasets used and/or analyzed during the current study are available from the corresponding author on reasonable request.

## Abbreviations

CD: Crohn's disease
CRP: C reactive protein
GERD: Gastro-esophageal reflux disease
GI: Gastro-intestinal
IBD: Inflammatory bowel disease
IBS: Irritable bowel syndrome
MUFA: Monounsaturated fatty acid
NAFLD: Non-alcoholic fatty liver disease
PRAL: Potential renal acid load
PUFA: Polyunsaturated fatty acid
SAFA: Saturated fatty acid
SCFAs: Short-chain fatty acids
TAC: Total antioxidant capacity
UC: Ulcerative colitis

## References

[1] Knowles SR, Graff LA, Wilding H, Hewitt C, Keefer L, Mikocka-Walus A (2018). Quality of life in inflammatory bowel disease: A systematic review and meta-analyses-part I. Inflamm. Bowel Dis..

[2] Rui W, Zhaoqi L, Shaojun L, Decai Z (2023). Global, regional and national burden of inflammatory bowel disease in 204 countries and territories from 1990 to 2019: A systematic analysis based on the Global Burden of Disease Study 2019. BMJ Open.

[3] Du L, Ha C (2020). Epidemiology and pathogenesis of ulcerative colitis. Gastroenterol. Clin..

[4] Yan, Y. Pathogenesis of inflammatory bowel diseases. in *Inflammatory Bowel Disease-Advances in Pathogenesis and Management* (IntechOpen, 2012).

[5] Hou JK, Abraham B, El-Serag H (2011). Dietary intake and risk of developing inflammatory bowel disease: A systematic review of the literature. J. Am. Coll. Gastroenterol..

[6] Keshteli AH, Madsen KL, Dieleman LA (2019). Diet in the pathogenesis and management of ulcerative colitis: A review of randomized controlled dietary interventions. Nutrients.

[7] Shivappa N, Hébert JR, Rashvand S, Rashidkhani B, Hekmatdoost A (2016). Inflammatory potential of diet and risk of ulcerative colitis in a case–control study from Iran. Nutr. Cancer.

[8] Karimi S, Tabataba-Vakili S, Yari Z, Alborzi F, Hedayati M, Ebrahimi-Daryani N (2019). The effects of two vitamin D regimens on ulcerative colitis activity index, quality of life and oxidant/anti-oxidant status. Nutr. J..

[9] Rahmani J, Kord-Varkaneh H, Hekmatdoost A, Thompson J, Clark C, Salehisahlabadi A (2019). Body mass index and risk of inflammatory bowel disease: A systematic review and dose-response meta-analysis of cohort studies of over a million participants. Obes. Rev..

[10] Antoniussen CS, Rasmussen HH, Holst M, Lauridsen C (2021). Reducing disease activity of inflammatory bowel disease by consumption of plant-based foods and nutrients. Front. Nutr..

[11] Thorburn AN, Macia L, Mackay CR (2014). Diet, metabolites, and “western-lifestyle” inflammatory diseases. Immunity.

[12] Spooren C, Pierik M, Zeegers M, Feskens E, Masclee A, Jonkers D (2013). The association of diet with onset and relapse in patients with inflammatory bowel disease. Aliment. Pharmacol. Ther..

[13] Hekmatdoost A, Wu X, Morampudi V, Innis SM, Jacobson K (2013). Dietary oils modify the host immune response and colonic tissue damage following *Citrobacter **rodentium* infection in mice. Am. J. Physiol. Gastrointest. Liver Physiol..

[14] Tayyem RF, Qalqili TR, Ajeen R, Rayyan YM (2021). Dietary patterns and the risk of inflammatory bowel disease: Findings from a case-control study. Nutrients.

[15] Hart AR, Luben R, Olsen A, Tjonneland A, Linseisen J, Nagel G (2008). Diet in the aetiology of ulcerative colitis: A European prospective cohort study. Digestion.

[16] Demetriou CA, Hadjisavvas A, Loizidou MA, Loucaides G, Neophytou I, Sieri S (2012). The mediterranean dietary pattern and breast cancer risk in Greek-Cypriot women: A case-control study. BMC Cancer.

[17] Estruch R (2010). Anti-inflammatory effects of the Mediterranean diet: The experience of the PREDIMED study. Proc. Nutr. Soc..

[18] Williams RS, Heilbronn LK, Chen DL, Coster AC, Greenfield JR, Samocha-Bonet D (2016). Dietary acid load, metabolic acidosis and insulin resistance: Lessons from cross-sectional and overfeeding studies in humans. Clin. Nutr..

[19] Williamson M, Moustaid-Moussa N, Gollahon L (2021). The molecular effects of dietary acid load on metabolic disease (the cellular PasaDoble: The fast-paced dance of pH regulation). Front. Mol. Med..

[20] Alam I, Alam I, Paracha PI, Pawelec G (2012). Higher estimates of daily dietary net endogenous acid production (NEAP) in the elderly as compared to the young in a healthy, free-living elderly population of Pakistan. Clin. Interv. Aging.

[21] Wu T, Seaver P, Lemus H, Hollenbach K, Wang E, Pierce JP (2019). Associations between dietary acid load and biomarkers of inflammation and hyperglycemia in breast cancer survivors. Nutrients.

[22] Osuna-Padilla I, Leal-Escobar G, Garza-García C, Rodríguez-Castellanos F (2019). Dietary acid load: Mechanisms and evidence of its health repercussions. Nefrologia.

[23] Osuna-Padilla IA, Leal-Escobar G, Garza-García CA, Rodríguez-Castellanos FE (2019). Dietary acid load: Mechanisms and evidence of its health repercussions. Nefrologia..

[24] Remer T, Dimitriou T, Manz F (2003). Dietary potential renal acid load and renal net acid excretion in healthy, free-living children and adolescents. Am. J. Clin. Nutr..

[25] Noormohammadi M, Eslamian G, Kazemi SN, Rashidkhani B (2022). Dietary acid load, alternative healthy eating index score, and bacterial vaginosis: is there any association? A case-control study. BMC Infect. Dis..

[26] Esche J, Krupp D, Mensink GB, Remer T (2018). Dietary potential renal acid load is positively associated with serum uric acid and odds of hyperuricemia in the German adult population. J. Nutr..

[27] Kellum JA, Song M, Almasri E (2006). Hyperchloremic acidosis increases circulating inflammatory molecules in experimental sepsis. Chest.

[28] Pedoto A, Caruso JE, Nandi J, Oler A, Hoffmann SP, Tassiopoulos AK (1999). Acidosis stimulates nitric oxide production and lung damage in rats. Am. J. Respir. Crit. Care Med..

[29] Pedoto A, Nandi J, Oler A, Camporesi EM, Hakim TS, Levine RA (2001). Role of nitric oxide in acidosis-induced intestinal injury in anesthetized rats. J. Lab. Clin. Med..

[30] Krupp D, Johner SA, Kalhoff H, Buyken AE, Remer T (2012). Long-term dietary potential renal acid load during adolescence is prospectively associated with indices of nonalcoholic fatty liver disease in young women. J. Nutr..

[31] Emamat H, Farhadnejad H, Poustchi H, Teymoori F, Bahrami A, Hekmatdoost A (2022). The association between dietary acid load and odds of non-alcoholic fatty liver disease: A case-control study. Nutr. Health..

[32] Emamat H, Tangestani H, Bahadoran Z, Khalili-Moghadam S, Mirmiran P (2019). The associations of dietary acid load with insulin resistance and type 2 diabetes: A systematic review of existing human studies. Recent Pat. Food Nutr. Agric..

[33] Banerjee T, Tucker K, Griswold M, Wyatt SB, Harman J, Young B (2018). Dietary potential renal acid load and risk of albuminuria and reduced kidney function in the Jackson Heart Study. J. Ren. Nutr..

[34] Bullo M, Amigó-Correig P, Márquez-Sandoval F, Babio N, Martínez-González MA, Estruch R (2009). Mediterranean diet and high dietary acid load associated with mixed nuts: Effect on bone metabolism in elderly subjects. J. Am. Geriatr. Soc..

[35] Rashvand S, Somi MH, Rashidkhani B, Hekmatdoost A (2015). Dietary fatty acid intakes are related to the risk of ulcerative colitis: A case–control study. Int. J. Colorectal Dis..

[36] Esfahani FH, Asghari G, Mirmiran P, Azizi F (2010). Reproducibility and relative validity of food group intake in a food frequency questionnaire developed for the Tehran Lipid and Glucose Study. J. Epidemiol..

[37] Gonick HC, Goldberg G, Mulcare D (1968). Reexamination of the acid-ash content of several diets. Am. J. Clin. Nutr..

[38] Scialla JJ, Anderson CA (2013). Dietary acid load: A novel nutritional target in chronic kidney disease?. Adv. Chronic Kidney Dis..

[39] Yan J, Wang L (2022). Dietary patterns and gut microbiota changes in inflammatory bowel disease: Current insights and future challenges. Nutrients.

[40] Peters V, Bolte L, Schuttert EM, Andreu-Sánchez S, Dijkstra G, Weersma RK (2022). Western and carnivorous dietary patterns are associated with greater likelihood of IBD development in a large prospective population-based cohort. Nutrients.

[41] Dong C, Chan SSM, Jantchou P, Racine A, Oldenburg B, Weiderpass E (2022). Meat intake is associated with a higher risk of ulcerative colitis in a large european prospective cohort studyø. Nutrients.

[42] Limketkai BN, Hamideh M, Shah R, Sauk JS, Jaffe N (2022). dietary patterns and their association with symptoms activity in inflammatory bowel diseases. Inflamm. Bowel Dis..

[43] Rahmani J, Kord-Varkaneh H, Ryan PM, Rashvand S, Clark C, Day AS (2019). Dietary total antioxidant capacity and risk of ulcerative colitis: A case-control study. J. Dig. Dis..

[44] Qiu P, Ishimoto T, Fu L, Zhang J, Zhang Z, Liu Y (2022). The gut microbiota in inflammatory bowel disease. Front. Cell Infect. Microbiol..

[45] Parada Venegas D, De La Fuente MK, Landskron G, González MJ, Quera R, Dijkstra G (2019). Short chain fatty acids (SCFAs)-mediated gut epithelial and immune regulation and its relevance for inflammatory bowel diseases. Front. Immunol..

[46] Bolte LA, Vila AV, Imhann F, Collij V, Gacesa R, Peters V (2021). Long-term dietary patterns are associated with pro-inflammatory and anti-inflammatory features of the gut microbiome. Gut.

[47] Jowett S, Seal C, Pearce M, Phillips E, Gregory W, Barton J (2004). Influence of dietary factors on the clinical course of ulcerative colitis: A prospective cohort study. Gut.

[48] Vidal-Lletjós S, Beaumont M, Tomé D, Benamouzig R, Blachier F, Lan A (2017). Dietary protein and amino acid supplementation in inflammatory bowel disease course: What impact on the colonic mucosa?. Nutrients.

[49] Blachier F, Beaumont M, Kim E (2019). Cysteine-derived hydrogen sulfide and gut health: A matter of endogenous or bacterial origin. Curr. Opin. Clin. Nutr. Metab. Care.

[50] Storz MA, Ronco AL (2022). Observational and clinical evidence that plant-based nutrition reduces dietary acid load. Curr. Opin. Clin. Nutr. Metab. Care.

[51] Kord Varkaneh H, Fatahi S, Rahmani J, Shab-Bidar S (2018). Association of dietary acid load with body composition and inflammatory biomarkers in patients with type 2 diabetes. MUQ J..

[52] Feuerstein, J. D. & Cheifetz, A. S. editors. Ulcerative colitis: epidemiology, diagnosis, and management. in *Mayo Clinic Proceedings* (Elsevier, 2014). 10.1016/j.mayocp.2014.07.002 25199861

[53] Cardoso H, Nunes ACR, Cruz A, Santos CC, Veloso FT (2006). Importance of serum cortisol levels in inflammatory bowel disease: 1197. Am. Coll. Gastroenterol..

